# Direct modelling of age standardized marginal relative survival through incorporation of time-dependent weights

**DOI:** 10.1186/s12874-021-01266-1

**Published:** 2021-04-24

**Authors:** Paul C. Lambert, Elisavet Syriopoulou, Mark R. Rutherford

**Affiliations:** 1 Biostatistics Research Group, Department of Health Sciences, University of Leicester, University Road, Leicester, UK; 2 Department of Medical Epidemiology and Biostatistics, Karolinska Institutet, Nobels Väg 12A, Stockholm, Sweden

**Keywords:** Net survival, Regression standardization, Relative survival

## Abstract

**Background:**

When quantifying the probability of survival in cancer patients using cancer registration data, it is common to estimate marginal relative survival, which under assumptions can be interpreted as marginal net survival. Net survival is a hypothetical construct giving the probability of being alive if it was only possible to die of the cancer under study, enabling comparisons between populations with differential mortality rates due to causes other the cancer under study. Marginal relative survival can be estimated non-parametrically (Pohar Perme estimator) or in a modeling framework. In a modeling framework, even when just interested in marginal relative survival it is necessary to model covariates that affect the expected mortality rates (e.g. age, sex and calendar year). The marginal relative survival function is then obtained through regression standardization. Given that these covariates will generally have non-proportional effects, the model can become complex before other exposure variables are even considered.

**Methods:**

We propose a flexible parametric model incorporating restricted cubic splines that directly estimates marginal relative survival and thus removes the need to model covariates that affect the expected mortality rates. In order to do this the likelihood needs to incorporate the marginal expected mortality rates at each event time taking account of informative censoring. In addition time-dependent weights are incorporated into the likelihood. An approximation is proposed through splitting the time scale into intervals, which enables the marginal relative survival model to be fitted using standard software. Additional weights can be incorporated when standardizing to an external reference population.

**Results:**

The methods are illustrated using national cancer registry data. In addition, a simulation study is performed to compare different estimators; a non-parametric approach, regression-standardization and the new marginal relative model. The simulations study shows the new approach is unbiased and has good relative precision compared to the non-parametric estimator.

**Conclusion:**

The approach enables estimation of standardized marginal relative survival without the need to model covariates that affect expected mortality rates and thus reduces the chance of model misspecification.

**Supplementary Information:**

The online version contains supplementary material available at (10.1186/s12874-021-01266-1).

## Background

When quantifying the probability of survival in cancer patients using cancer registration data it is common to estimate marginal relative survival, which under assumptions can be interpreted as marginal net survival [1, 2]. Net survival is a hypothetical construct giving the probability of being alive if it was only possible to die of the cancer under study. In these studies cause of death information is either unavailable or, more commonly, deemed unreliable [3, 4] and so expected mortality rates are incorporated so that mortality in excess of that expected can be estimated.

Relative survival is likely to vary by demographic factors such as age and sex, and disease severity, but is often reported as a marginal estimate giving the average survival in the study population. To ensure fair comparisons between population groups age-standardization is generally performed [5] where a common age distribution is forced on the groups being compared. The arguments for standardization could also apply to other covariates that vary between groups being compared.

Marginal relative survival can be estimated non-parametrically using the Pohar Perme estimator [6]. In order to age-standardize when using the Pohar Perme estimator it is usual to obtain separate estimates for different age groups, with the marginal estimate obtained through a weighted average of the age group specific effects. An alternative is to up or down weight individuals relative to a reference population [7, 8]. Standardization can also be performed using a modeling approach [9–11], which allows estimation of conditional effects and well as marginal estimates, through *regression standardization* [2, 12].

In a standard survival model, i.e. not in the relative survival framework,if one is just interested in an estimate of marginal survival, then it is not necessary to model any covariates. This means that for a reasonable parametric model with no covariates the estimated survival function should be in good agreement with the non-parametric Kaplan-Meier curve. However, in the relative survival framework there will be a difference between the estimated relative survival from a model with no covariates and a non-parametric (Pohar Perme) estimate. This is due to need to incorporate expected mortality rates that vary between individuals (by age, sex, calendar year etc) in the relative survival model. Thus, red when using a relative survival model to estimate, marginal relative survival, then covariates that affect expected mortality rates should be modelled so that the marginal effect can then be estimated through regression standardization [2, 12].

The need to model covariates that affect expected mortality rates, even when interest only lies in marginal relative survival, increases the likelihood of model misspecification, particularly as it is very common to have to model time-dependent effects (non-proportional excess hazards). In this paper we propose a model that directly estimates marginal relative survival and thus removes the need to model variables that reflect the variation in expected mortality rates. In the “Methods” section we define the conditional and marginal models and describe how incorporation of individual level weights allows external age standardization and enable covariates to be incorporated. The “Results” section includes a simulation study evaluating statistical properties of different estimators and illustrates the methods using an example of individuals diagnosed with melanoma. The paper is concluded with a discussion.

## Methods

In the relative survival framework it is assumed that the overall all-cause mortality rate, *h*(*t*|*X*_*i*_), for an individual with covariate pattern *X*_*i*_, is the sum of the expected mortality rate, *h*^∗^(*t*|*X*_*i*_), and the excess mortality rate, *λ*(*t*|*X*_*i*_).

1 $$ h(t|\pmb{X}_{i}) = h^{*}(t|\pmb{X}_{i}) + \lambda(t|\pmb{X}_{i})   $$

For simplicity it is assumed that covariates, *X*_*i*_, are the same for the expected and excess mortality rates, but this can be relaxed. Expected mortality rates are stratified by age, sex, calendar year and potentially other demographic covariates. The relative survival for covariate pattern *X*_*i*_ is,

$$ {R}(t|\pmb{X}_{i}) = \exp\left(-\int_{0}^{t}{\lambda(u|\pmb{X}_{i}) du}\right) $$

The marginal relative survival, *R*_*m*_(*t*|*X*), is the expectation over covariates, *X*, i.e. *E*_*X*_[*R*(*t*|*X*)]. This can be estimated in a modelling framework when incorporating these covariates, *X*, by averaging the individual estimates, $\widehat {R}(t|\pmb {X}_{i})$.

$$ \widehat{R}_{m}(t|\pmb{X}) = \frac{1}{N}\sum_{i=1}^{N}{\widehat{R}(t|\pmb{X}_{i})} $$

The marginal excess mortality rate function, *λ*_*m*_(*t*|*X*) can be obtained through the usual transformation from survival to hazard function, $h(t) = -\frac {d \ln [S(t)]}{dt}$.

2 $$  \lambda_{m}(t|\pmb{X}) = \frac{E_{\pmb{X}}\left[R(t|\pmb{X})\lambda(t|\pmb{X})\right]}{E_{\pmb{X}}[R(t|\pmb{X})]}  $$

and can be estimated in a parametric modeling framework when modeling covariates *X* by,

$$ \widehat{\lambda}_{m}(t|\pmb{X}) = \frac{\frac{1}{N}\sum_{i=1}^{N}{\widehat{R}(t|\pmb{X}_{i})\widehat{\lambda}(t|\pmb{X}_{i})}}{\frac{1}{N}\sum_{i=1}^{N}{\widehat{R}(t|\pmb{X}_{i})}} $$

### A conditional model with no covariates for the excess mortality rate

Consider the conditional model in Eq. (1) without including covariates *X*_*i*_ for the excess mortality rate.

3 $$  h(t|\pmb{X}_{i}) = h^{*}(t|\pmb{X}_{i}) + \lambda(t)  $$

This assumes that the excess mortality rate, *λ*(*t*), is the same for all individuals. This would mean that all-cause mortality rate would vary between individuals only due to variation in the expected mortality rates and not variation in excess mortality rates. This is different from that defined in Eq. (2) where the individual excess mortality rates vary between individuals.

### Likelihood

We adopt a fully parametric approach, so will model how excess mortality rates vary over time from diagnosis and by covariates. For an observed all-cause survival/censoring time, *t*_*i*_ and event indicator for death due to any cause, *d*_*i*_, the log-likelihood contribution of the *i*^*th*^ individual with covariate pattern, *X*_*i*_, for a relative survival model is

$$ \ln L_{i} = d_{i}\ln\left[h^{*}(t_{i}|\pmb{X}_{i}) + \lambda(t_{i}|\pmb{X}_{i},\pmb{\beta},\pmb{\gamma}) \right] - \Lambda(t_{i}|\pmb{X}_{i},\pmb{\beta},\pmb{\gamma}) $$

where *Λ*(*t*|*X*_*i*_,*β*,*γ*) is the cumulative excess mortality function with parameters, *β*, modelling covariate effects and, *γ*, modeling the effect of time from diagnosis [11, 13].

For a marginal model with no covariates the marginal excess mortality rate, *λ*_*m*_(*t*|*X*), as defined in Eq. (2), needs to be directly estimated; *X* thus denotes covariates that can impact on both expected and excess mortality rates. Rather than incorporate, *h*^∗^(*t*_*i*_|*X*_*i*_), the individual expected hazard for the *i*^*th*^ individual at time *t*_*i*_, a suitable estimate of the marginal expected mortality rate needs to be incorporated. A naive way to do this would be including the mean of *h*^∗^(*t*_*i*_|*X*_*i*_) among those at risk at time *t*_*i*_. However, net survival is defined in the hypothetical world where it is not possible to die from other causes, but it is estimated in the real world where individuals may die from both their cancer and from other causes. This means that with increasing time from diagnosis individuals with a higher risk of dying from other causes will be underrepresented. This should be taken into account in both the likelihood and when estimating the mean expected mortality rate. A similar idea to that proposed by Pohar Perme et al. [6] for the non-parametric estimator is used by upweighting by the inverse of the expected survival, *S*^∗^(*t*|*X*_*i*_), where,

$$S^{*}(t|\pmb{X}_{i}) = \exp\left(-\int_{0}^{t}{h^{*}(u|\pmb{X}_{i}) du}\right) $$

and defining the individual level, time-dependent weights $w_{i}^{*}(t)$ as

$$ w_{i}^{*}(t) = \frac{1}{S^{*}(t|\pmb{X}_{i})} $$

The mean expected mortality rate at time *t*_*i*_ incorporating weights, $w_{i}^{*}(t_{i})$, is

4 $$ \bar{h}^{*}(t_{i})=\frac{\sum\limits_{j\in \mathcal{R}(t_{i})} { w_{j}^{*}(t_{i}) h^{*}(t_{i}|\pmb{X}_{j})}}{\sum\limits_{j\in \mathcal{R}(t_{i})} { w_{j}^{*}(t_{i})}}   $$

where $\mathcal {R}(t_{i})$ is the set of those at risk at time *t*_*i*_.

The weighted marginal expected mortality rates, $\bar {h}^{*}(t_{i})$ can then be incorporated into the weighted likelihood rather than *h*^∗^(*t*_*i*_|*X*_*i*_) together with weights, $w_{i}^{*}(t_{i})$,

5 $$ \begin{aligned} \ln L_{i} &= d_{i} w_{i}^{*}(t_{i})\ln\left[\bar{h}^{*}(t_{i}) + \lambda_{m}(t_{i}|\pmb{\gamma}) \right] \\&\quad- \int_{0}^{t_{i}}{w_{i}^{*}(u)\lambda_{m}(u|\pmb{\gamma}) du}  \end{aligned}  $$

Note that Eq. (4) only needs to be calculated at event times and is not needed for individuals with censored times. The integral in Eq. (5) will generally not be analytically tractable. A numerical integration method, such as Gaussian quadrature, could be incorporated into the estimation process. However, we choose to split the time-scale into a number of intervals and assume that the weight is constant within each interval. The likelihood then becomes,

6 $$ \begin{aligned} \ln L_{i} &= d_{i} w_{i}^{*}(t_{i})\ln\left[\bar{h}^{*}(t_{i}) + \lambda_{m}(t_{i}|\pmb{\gamma}) \right] \\&\quad- \sum_{k=1}^{M_{i}} {w_{i}^{*}(t_{k})\left(\Lambda_{m}(t_{i(k)}|\pmb{\gamma}) - \Lambda_{m}(t_{i(k-1)}|\pmb{\gamma})\right)}  \end{aligned}  $$

where *M*_*i*_ is the number of intervals for the *i*^*th*^ subject. An advantage of this approach is that after splitting the time-scale and calculating the weights, standard parametric relative survival models can be used. This requires the software to incorporate both weights and left truncation into the likelihood. There needs to be a choice of how finely to split the time-scale. As the weights depend upon expected mortality rates, the weights will vary continuously, so a choice needs to be made at what point within the interval to calculate the weight. We use the mid-point of the interval [14]. More time intervals will result in greater precision, but increase computational time. The choice of the number of time intervals is investigated in the example in the “Results” section. The weights vary within individuals and leads to within-subject correlation. Therefore, a cluster robust sandwich estimator of the variance is used [15]. This is similar to other methods that use time-dependent weights, such as the Fine and Gray subhazard model [16] or the parametric equivalent [17].

### External age-standardization

In order to compare estimates of marginal relative survival between different population groups it is necessary to age-standardize to the same age distribution. In the non-parametric setting the usual approach is to estimate marginal relative survival separately within age groups and then obtain a weighted average of the age-specific estimates, with weights equal to the proportion within each age group in the reference population. In a modelling framework regression standardization is performed with each individual up or downweighted using the ratio of the proportion in the age group to which the individual belongs and the proportion in the reference age group [2]. A similar idea can be used within the marginal model that enables externally age-standardized estimates to be obtained without the need to model, or stratify by, age.

Let $p^{a}_{i}$ be the proportion in the age group to which the *i*^*th*^ individual belongs and $p^{R}_{i}$ be the corresponding proportion in the reference population. Weights can be defined to upweight or downweight individual relative to the reference population.

7 $$ w_{i}^{a} = \frac{p^{R}_{i}}{p^{a}_{i}}   $$

These weights can then be combined with the inverse expected survival weights,

$$ w_{i}(t) = w_{i}^{a} w_{i}^{*}(t) $$

These weights are the same as those defined by Sasieni and Brentnall [7] for use in non-parametric relative survival estimators. The weights need to be used when calculating $\bar {h}^{*}(t_{i})$ by substituting $w_{i}^{*}(t)$ for *w*_*i*_(*t*) in Eq. (4) and in the likelihood in Eq. (6). It is common just to standardize by age, but the approach is applicable when standardizing over multiple covariates.

### Modeling covariates

When the aim is to make contrasts between different population groups then covariates can be added to the marginal model. For example, these covariate could be different regions/countries, socio-economic groups, time-periods or sexes. As the age distribution may vary between the groups being compared it is important to age-standardize, but now the weights defined in Eq. (7) should be calculated separately within subgroups. In addition, $\bar {h}^{*}(t_{i})$ should be calculated separately within each subgroup.

### Choice of parametric model

The likelihood defined in Eq. (6) could be used for a variety of parametric models. Here we use flexible parametric survival models on the log-cumulative excess hazard scale [9] that incorporate restricted cubic splines to model the effect of time from diagnosis. An advantage of modeling on the log-cumulative excess hazard scale is that the it provides an analytical form for the cumulative excess hazard that is required for the likelihood in Eq. (6). The model for the log cumulative excess hazard, *Λ*(*t*|*k*_0_,*γ*), where *k*_0_ is a vector of knots and *γ* the associated splines parameters, is

$$\ln\left[\Lambda(t|\pmb{k_{0}},\pmb{\gamma})\right] = \eta(t|\pmb{k_{0}},\pmb{\gamma}) = s\left(\ln(t)|\pmb{k_{0}},\pmb{\gamma}\right) $$

where *s*(ln(*t*)|*k*_0_,*γ*) is a restricted cubic spline function of log time. The number of parameters to model the baseline is determined by the number of knots for the restricted cubic spine function with the number of parameters (including the intercept) being equal to the number of knots. Simulation studies have shown that the models give negligible bias when estimating survival functions across a wide range of scenarios [18, 19]. For more details on these models see Royston and Lambert [13].

After fitting a model the estimated marginal relative survival and marginal excess mortality functions can be estimated,

$$\begin{aligned} \widehat{R}_{m}(t|\pmb{X}) &= \exp[-\exp(\widehat{\eta}(t|\pmb{k_{0}},\pmb{\gamma}))] \ \ \ \ \ \ \ \\ \widehat{\lambda}_{m}(t|\pmb{X}) &= \frac{d s\left(\ln(t)|\pmb{k_{0}},\widehat{\pmb{\gamma}}\right)}{dt} \exp[\widehat{\eta}(t|\pmb{k_{0}},\pmb{\gamma})] \end{aligned} $$

In Appendix I, we describe how a semi-parametric marginal model could be fitted through estimation of a separate parameter for each event type using Poisson regression and how this is a equivalent to the Pohar Perme non-parametric estimate when not modeling covariates in order to demonstrate how the methods are related. However, we do not advocate this approach due to the computational intensity of splitting the time scale at unique time points and estimating a separate parameter for each time interval.

### Simulation study

A small simulation study was performed to quantify any potential bias in the different methods, the coverage probabilities and the variation in the estimates. We simulate two scenarios where there is substantial variation in relative survival by age, a situation which leads to higher bias for some methods [2]. In Scenario 1 the variation the excess hazard ratio decreases over time and in Scenario 2 we assume the effect of age is proportional over time. The simulation strategy is outlined below.

1. The sample size of each dataset was set at 1000.
2. All subjects were assumed to be male and diagnosed in one calendar year, 2009.
3. Age at diagnosis was sampled from a Normal distribution with mean 66 and standard deviation 13.
4. Times of death due to cancer was generated with the baseline survival (at the mean age) having a Weibull distribution with shape paramater, *γ*=0.5, and scale parameter, *λ*=0.2. The hazard function for the Weibull model is *λγt*^*γ*−1^.
5. The effect of age at diagnosis (*agediag*) varied between the 2 scenarios. Scenario 1 *agediag* was assumed to be non-proportional over time with an excess hazard ratio of exp(0.1−0.01(*agediag*−66)*t*). The time-dependent excess hazard ratios and relative survival for selected ages are shown in Fig. 1. Note that the excess hazard ratios get closer to the null as time increases. Scenario 2 *agediag* was assumed to be non-proportional over time with an excess hazard ratio of exp(0.03(*agediag*−66)). The excess hazard ratios and relative survival for selected ages are shown in Fig. 1.

**Fig. 1:**
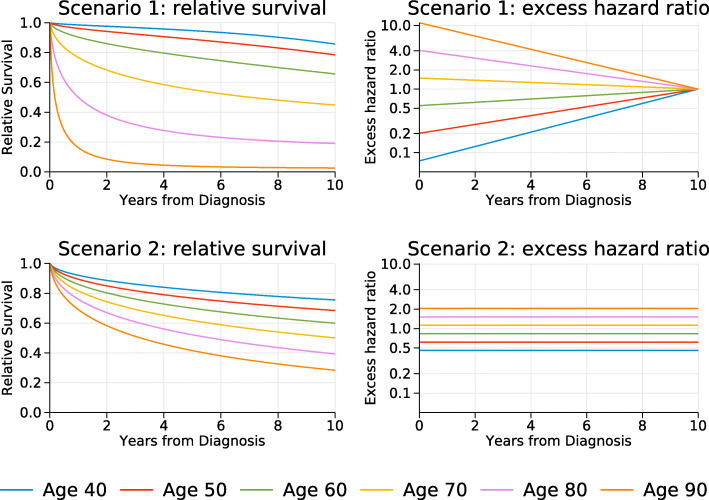
True relative survival and excess hazard ratios for selected ages. For the excess hazard ratios the mean age at diagnosis, 66, is the reference
6. Times to death from other causes were generated from exponential distributions with mortality rates obtained from expected population mortality rates in England in 2009 [20]. A different rate was used for each year of follow to account for the fact that attained age was increasing. If the simulated value was greater than 1 for any yearly interval then the individual was assumed to be alive at the start of the next interval. If the value was less than one, it was assumed that the individual died in the interval.
7. The observed time to death was taken as the minimum of the cancer specific time to death and the other cause time to death.
8. The following analyses were performed on each simulated data set. All flexible parametric models used 6 knots to model the effect of follow-up time.
  a The non-parametric Pohar Perme method to estimate marginal relative survival [6].
  b A conditional flexible parametric relative survival model with no-covariates, see Eq. (3).
  c A conditional flexible parametric relative survival model fitting a linear effect of age and assuming proportional hazards, see Eq. (1). The marginal relative survival was estimated using regression standardization.
  d A conditional flexible parametric relative survival model with the effect of age modelled using restricted cubic splines (4 knots) and allowing for non-proportional hazards with an interaction between the age splines and spines variables to model the effect of follow-up time, see Eq. (1). Marginal relative survival was then estimated using regression standardization.
  e A flexible parametric marginal model to directly estimate marginal relative survival. The time-scale was split every 0.2 years to incorporate the weights.
9. 1000 datasets were generated. In estimation of the coverage this gives a standard error of $100\sqrt (0.95*0.05/1000) = 0.69$ percentage points.
10. For each method the bias, the mean square error (MSE), the coverage and relative percentage increase in precision compared to the non-parametric Pohar Perme estimator at 1, 5 and 10 years post diagnosis was reported.

## Results

### Simulation study

The results of the simulation study for scenarios 1 and 2 are shown in Tables 1 and 2 respectively. In both scenarios bias is negligible for the Pohar Perme method, the marginal model and regression standardization when fitting a model with time-dependent effects for age with coverage close to 95%. Regression standardization when assuming proportional hazards gives a bias of 0.4, 0.9 and 2.0 percentage points at 1, 5 and 10 years respectively for Scenario 1, illustrating the potential issue of fitting an incorrect model. In Scenario 2 bias was lower as the variation in excess mortality reduced as a function of follow-up time [2].

**Table 1:** Bias (bold font), coverage (italics font) and MSE (normal font) for simulation scenario 1 comparing the non parametric Pohar Perme estimator, a conditional model (without covariates), regression standardization under proportional hazards (PH), regression standardization under non-proportional hazards (Non PH) and a marginal model. Bias is expressed as a difference in probabilities

	Years from diagnosis)		
Method	1	5	10
Pohar Perme	**0.0005**	**0.0006**	**0.0006**
	*95.4*	*95.1*	*95.4*
	198.676	302.290	430.220
Conditional Model	**0.0261**	**0.0486**	**0.0457**
	*53.6*	*19.5*	*29.3*
	873.184	2653.730	2450.234
Regression standardization (PH)	**-0.0044**	**-0.0093**	**-0.0202**
	*92.5*	*93.5*	*80.7*
	204.942	353.266	737.678
Regression standardization (Non PH) ^∗^	**0.0025**	**0.0016**	**-0.0006**
	*93.6*	*97.2*	*96.5*
	188.909	283.351	381.325
Marginal model	**0.0032**	**0.0020**	**0.0018**
	*95.7*	*95.7*	*95.5*
	192.043	288.790	420.932
Relative % increase in precision ^+^			
Regression standardization (PH)	7.2	12.9	30.0
Regression standardization (Non PH) ^∗^	8.6	7.6	12.9
Marginal model	9.0	6.0	3.0

**bias**, *Coverage*, MSE

^∗^ 0.5% of models did not converge

^+^ compared to Pohar Perme

PH - proportional hazards

Non PH - non proportional hazards

**Table 2:** Bias (bold font), coverage (italics font) and MSE (normal font) for simulation scenario 2 comparing the non parametric Pohar Perme estimator, a conditional model (without covariates), regression standardization under proportional hazards (PH), regression standardization under non-proportional hazards (Non PH) and a marginal model. Bias is expressed as a difference in probabilities

	Years from diagnosis)		
Method	1	5	10
Pohar Perme	**0.0001**	**-0.0000**	**0.0005**
	*94.6*	*95.1*	*95.0*
	180.679	416.913	936.228
Conditional Model	**0.0092**	**0.0274**	**0.0434**
	*87.8*	*61.6*	*34.9*
	235.224	1052.450	2264.524
Regression standardization (PH)	**0.0002**	**-0.0000**	**0.0008**
	*88.9*	*96.1*	*94.7*
	154.728	313.687	395.280
Regression standardization (Non PH) ^∗^	**-0.0001**	**0.0006**	**0.0022**
	*88.6*	*96.1*	*96.7*
	158.038	370.164	630.970
Marginal model	**-0.0003**	**0.0011**	**0.0046**
	*95.5*	*95.0*	*93.5*
	151.551	374.557	888.256
Relative % increase in precision ^+^			
Regression standardization (PH)	16.8	32.9	137.1
Regression standardization (Non PH) ^∗^	14.3	12.7	49.4
Marginal model	19.3	11.7	8.0

**bias**, *Coverage*, MSE

^∗^ 18.3% of models did not converge

^+^ compared to Pohar Perme

PH - proportional hazards

Non PH - non proportional hazards

Fitting a conditional survival model with no covariates gives a bias of 2.6, 4.9 and 4.6 percentage points at 1, 5 and 10 years respectively for Scenario 1, with bias of 0.0, 2.7 and 4.3 percentage points for Scenario 2.

The marginal relative survival model has a lower MSE at all times when compared to the Pohar Perme method for both scenarios. Regression standardization of the model allowing a non-proportional effect of age has similar MSE to the marginal model at 1 and 5 years, but a lower MSE at 10 years.

Regression standardization with non-proportional hazards and the marginal model had similar relative increases in precision when compared to the Pohar Perme estimator at 1 and 5 years. At 10 years, the marginal model had higher relative precision than the Pohar Perme method, but the increase was notably greater when using regression standardization.

Five (0.5*%*) of the models with time-dependent effect of age in Scenario 1 and 183 (18.3*%*) in Scenario 2 failed to converge indicating a potential problem in using complex models to model the non-proportional effects of age when there are very few individuals still at risk towards the end of follow-up.

In summary, the simulation study shows the marginal model has negligible bias and greater precision than the non-parametric Pohar Perme estimator.

### Application to melanoma example

The methods are illustrated using data on 4,744 patients diagnosed with melanoma (a type of skin cancer) in a Northern European country between 1985–1994. The data is from a national cancer registry and so attempts to capture all diagnosed cases of Melanoma in the country. These data are distributed with the Stata strs package [21]. The code to implement the methods are shown in Appendix II with datasets freely available.

**Fig. 2:**
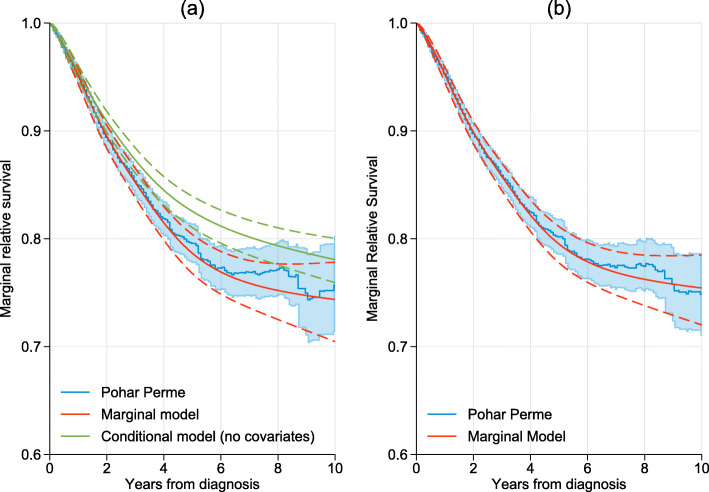
Estimates of marginal relative survival: Panel (**a**) shows internally age-standardized estimates using the non-parametric Pohar Perme method and the marginal relative survival model. Also shown is the estimate from the conditional relative survival model with no-covariates. Panel (**b**) shows externally age-standardized estimates from the non-parametric Pohar Perme method and marginal relative survival model. 95% confidence intervals are shown by either dashed lines or the shaded areas

Figure 2a shows the non-parametric Pohar Perme estimator together with the estimated modeled marginal relative survival. These are internally age standardized estimates as the expectation is over the observed age distribution. Here a model with 6 knots (i.e. five restricted cubic spline variables) were used to model the marginal log cumulative excess hazard. The time-scale was split every 0.2 years when incorporating the weights. Agreement can be seen to be very good. A feature of the Pohar Perme estimator is that it is highly variable towards the end of follow-up. This is due to the impact of the weights, with the small number of older individuals who are still alive having high influence. Also shown on this figure is the conditional model with no-covariates which shows a clear difference when compared to the other methods.

Figure 2b shows the externally age-standardized marginal relative survival using Pohar Perme and the marginal relative survival model. The age groups were <45,45−54,55−64,65−74,75+ years of age with standardization weights as defined in the International Cancer Survival Standard (ICSS) [5]. In order to age-standardize using the Pohar-Perme estimator separate estimates for each age group were obtained and then a weighted average was calculated. The marginal relative survival model does not need to stratify or model the effect of age due to the incorporation of the weights defined in Eq. (7). The Figure shows that, in this example, external age-standardization makes little difference when compared to the internally age-standardized estimates in Fig. 2a as the reference age-distribution was similar to that observed in the study population.

In Fig. 2 the time-scale was split every 0.2 years with the weights taken at the midpoint of each interval and 6 knots (5 spline variables) were used to model the baseline. Table 3 shows the estimated survival at 1, 5 and 10 years using a variety of time splits, at 0.05, 0.1, 0.2, 0.5, 1 and 2.5 years, and various number of knots (4, 6, 8 and 10). There is very little difference in any of the estimates at 1-year with the largest difference, 0.002. With increasing time there is more variation, but for time-splits from 0.05 to 0.2 years yields a difference of 0.002 or less at 10 years. Larger time-splits lead to larger differences, which is not surprising as the weights are forced to be constant within each interval.

**Table 3:** Estimated marginal relative survival at 1, 5 and 10 years from diagnosis using time splits at 0.05, 0.1, 0.2, 0.5, 1 and 2.5 years and using 4 6 8 and 10 knots to model the baseline

Time	Split	4 knots	5 knots	6 knots	7 knots
1 years	0.05	**0.951** (0.943,0.958)	**0.951** (0.943,0.958)	**0.951** (0.943,0.958)	**0.952** (0.944,0.958)
	0.1	**0.951** (0.943,0.958)	**0.951** (0.943,0.958)	**0.951** (0.943,0.958)	**0.952** (0.944,0.959)
	0.2	**0.951** (0.943,0.958)	**0.951** (0.943,0.958)	**0.951** (0.943,0.958)	**0.952** (0.944,0.959)
	0.5	**0.952** (0.944,0.958)	**0.952** (0.944,0.959)	**0.952** (0.944,0.958)	**0.952** (0.945,0.959)
	1	**0.952** (0.944,0.959)	**0.952** (0.944,0.959)	**0.952** (0.944,0.959)	**0.953** (0.945,0.960)
	2.5	**0.954** (0.946,0.960)	**0.954** (0.946,0.961)	**0.954** (0.946,0.960)	**0.955** (0.947,0.961)
5 years	0.05	**0.787** (0.769,0.805)	**0.786** (0.767,0.803)	**0.786** (0.767,0.803)	**0.786** (0.767,0.803)
	0.1	**0.788** (0.769,0.805)	**0.786** (0.767,0.803)	**0.786** (0.767,0.804)	**0.786** (0.767,0.803)
	0.2	**0.788** (0.770,0.806)	**0.787** (0.768,0.804)	**0.787** (0.768,0.804)	**0.787** (0.768,0.804)
	0.5	**0.790** (0.772,0.807)	**0.789** (0.770,0.806)	**0.789** (0.770,0.806)	**0.789** (0.770,0.806)
	1	**0.793** (0.775,0.810)	**0.792** (0.773,0.809)	**0.792** (0.773,0.809)	**0.792** (0.773,0.809)
	2.5	**0.803** (0.786,0.820)	**0.801** (0.783,0.818)	**0.801** (0.783,0.818)	**0.801** (0.783,0.818)
10 years	0.05	**0.737** (0.700,0.770)	**0.742** (0.703,0.777)	**0.743** (0.703,0.779)	**0.743** (0.701,0.780)
	0.1	**0.737** (0.700,0.771)	**0.742** (0.703,0.777)	**0.744** (0.703,0.780)	**0.744** (0.702,0.780)
	0.2	**0.739** (0.702,0.772)	**0.744** (0.705,0.778)	**0.745** (0.705,0.781)	**0.745** (0.703,0.782)
	0.5	**0.742** (0.706,0.775)	**0.747** (0.709,0.782)	**0.749** (0.709,0.784)	**0.749** (0.708,0.785)
	1	**0.748** (0.712,0.780)	**0.753** (0.715,0.786)	**0.754** (0.716,0.789)	**0.754** (0.714,0.789)
	2.5	**0.767** (0.734,0.796)	**0.772** (0.738,0.803)	**0.774** (0.739,0.806)	**0.774** (0.738,0.806)

Point estimates are in bold with 95% confidence intervals in braces

Point estimates shown in bold font and 95% confidence intervals in normal font

If the mean expected mortality defined in Eq. (4) is calculated separately for males and females then the effect of sex can be modelled. When doing so one will generally want to age-standardize as the age distributions of the groups being compared could differ. There is a choice here about what age distribution to standardize to. This could be the ICSS weights used above, which would allow comparisons with other studies using the same age standard. Alternatively, if measures more relevant to the study population are required then this could be the joint age distribution of male and females or the age distribution of one of the groups. Here the age distribution of males will be used to illustrate some useful interpretations.

**Fig. 3:**
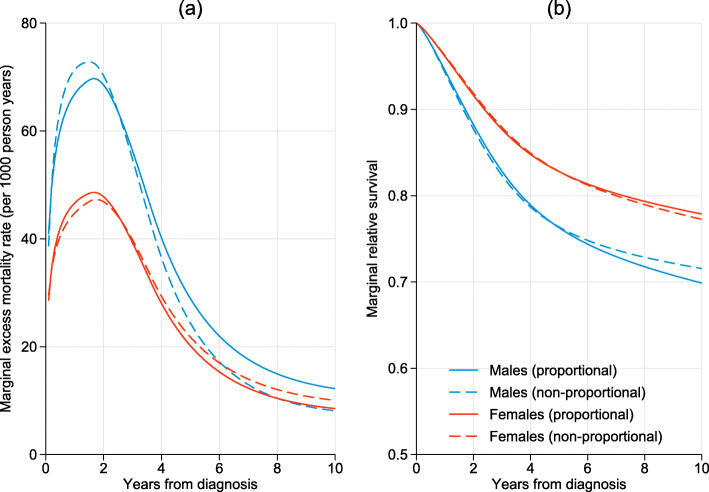
Estimated marginal excess mortality rate (panel (**a**)) and marginal relative survival functions (panel (**b**)) for proportional and non-proportional models. Estimates have been standardized to the age distribution of the males

It could be simplistically assumed that the marginal excess mortality rates are proportional. Fitting such a model yields a marginal excess mortality rate ratio for females compared to males of 0.70 (95% CI: 0.59 to 0.83). The assumption of proportional marginal hazards can be relaxed by incorporating an interaction between the effect of being female and the effect of time. This was done using a spline term with 4 knots, i.e. three additional parameters. The excess mortality rates for both the proportional hazards model and non-proportional model are shown in Fig. 3a with the corresponding marginal relative survival functions in Fig. 3b. The curves for each sex are very similar as the proportionality assumption appears reasonable here; (a decrease in log-likelihood of 0.71 with 3 degrees of freedom).

As we have standardized each of these estimates to the age distribution of males the curves give the factual estimates for males, i.e. given their own age distribution and the counterfactual effect for males if they had the same relative survival as females. This is because the age-distribution for males is being applied to the effect of females.

**Fig. 4:**
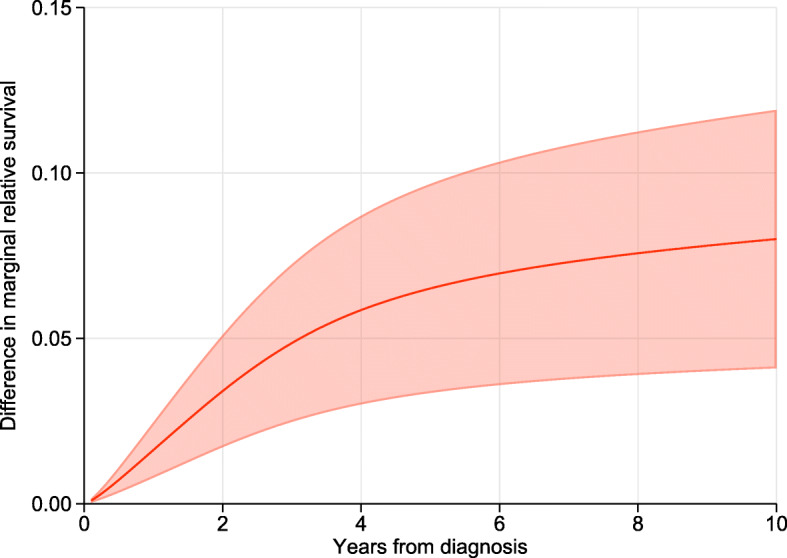
Difference in marginal relative survival with 95% confidence interval (shaded area) comparing females to males

Figure 4 shows the difference in the two marginal relative survival functions with 95% confidence intervals. As the age distribution of males has been used as the age reference, this gives an estimate how much the marginal relative survival would increase if males had the same relative survival as females.

## Discussion

We have demonstrated how marginal estimates of relative survival can be directly modeled through calculation of appropriately weighted mean expected mortality rates and introducing weights into the likelihood. We have also shown how additional weights can be included so that age-standardized marginal relative survival is directly estimated without the need to model or stratify by age. In addition we have shown that contrasts of marginal relative survival can be directly estimated from the marginal model. Although the relative survival framework has almost exclusively used within cancer, the methods described here could be utilized in other disease areas [22, 23].

When making comparisons between population groups there is generally a need to age-standardize as in most situations there will be a difference in the age distribution and there is usually a strong relationship between age and relative survival. Introducing individual level relative weights enables external age-standardization without the need to stratify or model age. Rutherford *et al* recently advocated the use of individual relative weights for the non-parametric Pohar Perme estimator for age standardization when data were sparse [8]. An important choice when age standardizing is in the age distribution used. Using ICSS weights allows comparisons with many other studies using the same weights, but using the age-distribution observed in the study population may be more useful for generalizing to a particular population. As separate effects do not need to be calculated by age group the use of narrower categories of age for the relative weights can be used. For example, in practice the oldest age group when standardizing for most cancer types is 75+. There can be substantial variation in relative survival within this age group and the age distribution within this age group may vary between population groups. Using the weighting approach proposed here one could, for example, apply the five year age groups presented by Corazziari et al. [5].

If just an overall summary of marginal relative survival is needed then the non-parametric Pohar Perme estimate could be used. An advantage of the marginal model is that it has negligible bias, but higher relative precision than then non-parametric approach. Our approach is particularly useful for comparisons between different population groups.

A limitation of the method is that the user has to choose the number of knots for the restricted cubic splines. Previous work has demonstrated that estimates are insensitive to the number of knots, as long as their are enough to capture the underlying shape of the hazard functions [18, 19, 24]. However, a sensitivity analysis, such as that displayed in Table 3 can be reassuring to both the analyst and others. The marginal model approximates the integral defined in the likelihood by splitting the time-scale (Eq. 6). The sensitivity analysis shown here shows that as long as the width of the intervals is at 0.2 years or less there is very little impact on the estimates, but smaller intervals would lead to improved accuracy. Although more accurate estimation could be obtained through calculating the integral numerically for each individual in the estimation process, we prefer the split time approach as it enables standard software to be used together with a host of useful post-estimation prediction commands.

In observational studies with time-to-event outcomes it is common to use inverse probability weighting (IPW) to adjust for confounders [25]. However, a conditional relative survival model without covariates gives a biased estimate of the marginal survival function (Fig. 2) and thus the IPW approach in not directly transferable to the relative survival framework. However, applying the IPW weights to the marginal model will enable the IPW approach to be used in the relative survival framework and this is being further investigated in ongoing work.

When there is interest in more detailed comparisons between population groups then a conditional model should be used. For example, to quantify how differences in relative survival vary by age. However, when there is only interest in the marginal relative survival and associated contrasts, the model proposed here simplifies the modelling process, has negligible bias and increased precision over the non parametric Pohar Perme estimate.

## Conclusion

When only marginal effects are of interest the model described here is particularly useful as it less prone to model misspecification as covariates that are associated with expected mortality rates do not need to be incorporated into the model.

## Software

Stata code to set-up data and fit the models for the melanoma example is shown in Appendix II of the supplementary material.

## Supplementary Information

**Additional file 1** Poisson regression and the Pohar Perme estimator.

**Additional file 2** Stata implementation of the models.

## Data Availability

The melanoma data used in this example is available in the strs Stata package (see reference 16). Code to fit the models is available in the supplementary material. Declarations

## Abbreviations

ICSS: International cancer survival standard
IPW: Inverse probability weighting
MSE: Mean squared error
