# Supplementary material for: Direct modelling of age standardized marginal relative survival through incorporation of time-dependent weights
Source: BMC Med Res Methodol. 2021 Apr 24;21:84. doi: 10.1186/s12874-021-01266-1 (PMC8070293; doi:10.1186/s12874-021-01266-1)
Supplement: Supplementary file 1 — Additional file 1 Poisson regression and the Pohar Perme estimator. [file 12874_2021_1266_MOESM1_ESM.pdf]

Appendix I Poisson Model

It is well known that a Cox model can fitted within the GLM framework using a Poisson model (1; 2) and such a model without covariates gives parameter estimates that are identical to the contribution of each unique time to the Nelson-Aalen estimate of the cumulative hazard. However, the approach is not a computationally efficient way to estimate such a model. Here we show that Poisson regression with appropriate weights is identical to Pohar Perme estimate. We are not advocating such an approach due computational reasons, but include details to demonstrate the link between the parametric and non-parametric estimators.

In order to fit the Poisson model the time-scale needs to be split at the unique failure times. This leads to each individual having as many rows of data that they are at risk for. Let d_{ij} be the event indicator for the ith subject in the jth time interval with t_j the time at the end of the interval. Note that d_{ij} will only take the value 1 for the final interval and only if the subject had an event. Let y_j denote the time at risk for the jth interval. In addition, define weights w_{ij}^* as,

$$w_{ij}^* = \frac{1}{S_{ij}^*(t_j)}$$

and the weighted mean hazard defined as in equation (4).

A Poisson model can be fitted with outcome d_{ij}, weights w_{ij}^* and offset $y_j\bar{h}^*(t_j)$, an identity link and indicator variables for each unique event time. Note that the offset gives the (weighted) expected number of deaths at the jth event time and y_j is the time from the previous event time (or from zero for the first event). A parameter, λ_j is estimated for each of the J unique death times. The contribution to the likelihood of the jth interval is the sum over the individuals at risk at time t_j.

$$\ln L_j = \sum_{i \in \mathcal{R}(t_j)} d_i w_{ij}^* \ln\left[\bar{h}^*(t_j) + \lambda_j\right] - w_{ij}^* \lambda_j y_j$$

where $\bar{h}^*(t_j)$ is defined in equation (4).

To obtain the maximum likelihood estimate, differentiate with respect to λ_j, set to zero and solve for λ_j.

$$\sum_{i \in \mathcal{R}(t_j)} \frac{d_{ij} w_{ij}^*}{\bar{h}^*(t_j) + \lambda_j} - \sum_{i \in \mathcal{R}(t_j)} w_{ij}^* y_j = 0$$

$$\sum_{i \in \mathcal{R}(t_j)} d_{ij} w_{ij}^* = y_j(\bar{h}^*(t_j) + \lambda_j) \sum_{i \in \mathcal{R}(t_j)} w_{ij}^*$$

$$\frac{\sum_{i \in \mathcal{R}(t_j)} d_{ij} w_{ij}^*}{\sum_{i \in \mathcal{R}(t_j)} w_{ij}^*} - \bar{h}^*(t_j) y_j = y_j \lambda_j$$

Substituting in the RHS of equation (**??**) for $\bar{h}^*(t_j)$ gives

$$\frac{\sum_{i \in \mathcal{R}(t_j)} d_{ij} w^*_{ij}}{\sum_{i \in \mathcal{R}(t_j)} w^*_{ij}} - \frac{\sum\limits_{i \in \mathcal{R}(t_j)} w^*_{ij} h^*(t_j)}{\sum\limits_{i \in \mathcal{R}(t_j)} w^*_{ij}} = y_j \lambda_j$$

The LHS is equivalent to the contribution of the jth event time for the change in the cumulative excess hazard for the Pohar Perme estimator.

The Stata code below demonstrates the equivalence using the first simulated dataset from the simulation study.

```
// Use first 250 observations of first simulated dataset
use scenario2_1 if _n<=250, clear

// declare survival data
stset t,f(dead) id(id) exit(time 10.5)

// Pohar Perme estimate
// Use fh (Fleming-Harrington) option for equivalence to poisson approach //

stpp R_pp using lifetab.dta, agediag(agediag) datediag(diagdate) ///
pmother(sex) fh

// split time scale at failure times
stsplit, at(failures) riskset(interval)
sort id (_t)

// attained age and calendar year
gen _year = year(diagdate + _t0*365.24)
gen _age  = min(floor(agediag + _t0),99)

// merge in expected rates
merge m:1 _year sex _age using lifetab, keep(match master)

// time at risk
gen double y = _t-_t0

// expected survival for each individival
bysort id (_t): gen double expsurv = exp(-sum(rate*y))
bysort _t _d:   gen lastobs = _n==_N & _d==1

// estimate marginal expected rates
gen double wt = 1/expsurv
bysort _t: egen double Y_w      = total(wt)
bysort _t: egen double hbar_num = total(rate/expsurv)
gen double hbar =  hbar_num/Y_w
```

```
// expected deaths
gen double d_star = hbar*y

// Poisson Model
glm _d ibn.interval [iweight=wt], family(poisson) link(identity) nocons offset(d_star) vce(c

// predict contribution of jth interval and sum over intervals
sort _t lastobs
predictnl double dLambda = sum(predict(xb nooffset)) if lastobs, ci(dLambda_lci dLambda_uci)

gen double R_pois = exp(-(dLambda))
gen double R_pois_lci = exp(-(dLambda_uci))
gen double R_pois_uci = exp(-(dLambda_lci))

// Check agreement between Pohar Perme and model based estimate.
assert reldif(R_pois,R_pp)<1e-08 if lastobs
assert reldif(R_pois_lci,R_pp_lci)<1e-04 if lastobs
assert reldif(R_pois_uci,R_pp_uci)<1e-04 if lastobs
```

We stress that we do not advocate this as a sensible way to estimate marginal relative survival using the Pohar Perme method, but want to illustrate the relationship between the methods. The code is slow on 250 observations and would be unfeasible when there are tens of thousands of observations.

References

[1] Whitehead J. Fitting Cox's regression model to survival data using GLIM. Applied Statistics. 1980;29:268–275.

[2] Laird N, Olivier D. Covariance analysis of censored survival data using log-linear analysis techniques. Journal of the American Statistical Association. 1981;76:231–240.
