# Supplementary material for: Direct modelling of age standardized marginal relative survival through incorporation of time-dependent weights
Source: BMC Med Res Methodol. 2021 Apr 24;21:84. doi: 10.1186/s12874-021-01266-1 (PMC8070293; doi:10.1186/s12874-021-01266-1)
Supplement: Supplementary file 2 — Additional file 2 Stata implementation of the models. [file 12874_2021_1266_MOESM2_ESM.pdf]

Appendix II: Implementation in Stata

```
use melanoma if year8594 == 1
stset surv_mm, failure(status=1,2) scale(12) exit(time 120) id(id)

// Pohar Perme estimate
stpp R_pp using popmort.dta, agediag(age) datediag(dx) pmother(sex)

// conditional model (no covariates)
gen _age = floor(min(age + _t,99))
gen _year = floor(year(dx + _t*365.24))

merge m:1 _age _year sex using popmort, keep(match master)
stpm2, scale(hazard) df (5) bhazard(rate)
range tt_cond 0 10 101
predict s_cond, surv timevar(tt) ci

preserve
rename _t PP_t
keep tt_cond s_cond* R_pp* PP_t
save cond, replace
restore

// marginal model
// setup data
mrsprep using popmort.dta, pmother(sex) agediag(age) datediag(dx) ///
                           pmmaxyear(2000)                        ///
                           verbose breaks(0(0.2)10)

// fit model
stset tstop [iw=wt], enter(tstart) failure(event==1)
stpm2, scale(hazard) df (5) bhazard(meanhazard_wt) vce(cluster id)
range tt_mrs 0 10 101
predict s_mrs, surv timevar(tt_mrs) ci

// merge in PP and conditional model
merge 1:1 _n using cond,  nogenerate

// Plot results
twoway (rarea R_pp_lci R_pp_uci  PP_t, sort connect(stairstep) color(${C1ci}))    ///
       (line R_pp PP_t, sort connect(stairstep) color(${C1}))                     ///
       (line s_mrs* tt_mrs, color(${C2} ${C2} ${C2}) lpattern(solid dash dash))   ///
       (line s_cond* tt_cond, color(${C3} ${C3} ${C3}) lpattern(solid dash dash)) ///
       , legend(order(2 "Pohar Perme" 3 "Marginal model"                          ///
               6 "Conditional model (no covariates)" )                            ///
               ring(0) cols(1) pos(7))                                            ///
       ylabel(0.6(0.1)1, format(%3.1f))                                           ///
       title("(a)")                                                               ///
       ytitle("Marginal relative survival")                                       ///
       xtitle("Years from diagnosis")                                             ///
       name(int_stand, replace)

//////////////////////////////
// External age standardization
//////////////////////////////
frame change default
drop _age _year rate prob

// change age groups to those defined in ICSS
drop agegrp
egen agegrp=cut(age), at(0  45 55 65 75 200) icodes
replace agegrp = agegrp + 1
label variable agegrp "Age group"
label define agegrplab 1 "0-44" 2 "45-54" 3 "55-64" 4 "65-74" 5 "75+", replace
label values agegrp agegrplab

recode agegrp (1=0.28) (2=0.17) (3=0.21) (4=0.20) (5=0.14), gen(ICSSwt)
```

```
//  Pohar Perme
stpp R_pp2 using popmort.dta, agediag(age) datediag(dx) pmother(sex) ///
                             standstrata(agegrp)                     ///
                             standweights(0.28 0.17 0.21 0.20 0.14)

preserve
rename _t PP_t
keep PP_t R_pp2*
save stand, replace
restore

// Proportion within each age group
local total= _N
bysort agegrp: gen a_age = _N/`total'
gen double wt_age = ICSSwt/a_age

// Prepare data for marginal model
mrsprep using popmort.dta, pmother(sex) agediag(age) datediag(dx) ///
                           pmmaxyear(2000)                        ///
                           verbose breaks(0(0.2)10)               ///
                           indweights(wt_age)                     ///
                           newframe(mrs_stand, replace)

// Fit model
stset tstop [iw=wt], enter(tstart) failure(event==1)
stpm2, scale(hazard) df(5) bhazard(meanhazard_wt) vce(cluster id)
range tt_stand 0 10 101
predict s_extstand, surv timevar(tt_stand) ci

// merge in PP
merge 1:1 _n using stand.dta

// Plot results
twoway (rarea R_pp2_lci R_pp2_uci PP_t, connect(stairstep) color(${C1ci})) ///
       (line R_pp2 PP_t, sort connect(stairstep) color(${C1})) ///
       (line s_extstand* tt_stand, sort color(${C2} ${C2} ${C2}) lpattern(solid dash dash)) ///
       , legend(order(2 "Pohar Perme" 3 "Marginal Model") ///
       ring(0) cols(1) pos(7)) ///
       ylabel(0.6(0.1)1, angle(h) format(%3.1f)) ///
       xtitle("Years from diagnosis") ///
       ytitle("Marginal Relative Survival") ///
       title("(b)") ///
       name(ext_stand, replace)

graph combine int_stand ext_stand, nocopies
```
